# Serum-Free Medium Enhances the Therapeutic Effects of Umbilical Cord Mesenchymal Stromal Cells on a Murine Model for Acute Colitis

**DOI:** 10.3389/fbioe.2020.00586

**Published:** 2020-06-26

**Authors:** Xiaoyun Wu, Daocheng Wu, Yongxu Mu, Yuxia Zhao, Zhijie Ma

**Affiliations:** ^1^Department of Pharmacy, Beijing Friendship Hospital, Capital Medical University, Beijing, China; ^2^Key Laboratory of Biomedical Information Engineering of the Ministry of Education, School of Life Science and Technology, Xi'an Jiaotong University, Xi'an, China; ^3^Interventional Department, The First Affiliated Hospital of Baotou Medical College, Inner Mongolia University of Science and Technology, Baotou, China; ^4^Department of Technology, Stem Cell Medicine Engineering & Technology Research Center of Inner Mongolia, Huhhot, China; ^5^Department of Blood, The People's Hospital of Xing'an League, Ulanhot, China

**Keywords:** mesenchymal stromal cells, colitis, therapeutic effect, mechanism, serum free, macrophage polarization

## Abstract

The usage of animal serum may ultimately prevent the application of *ex vivo* cultured mesenchymal stromal cells (MSCs) in a clinical setting due to safety concerns and batch-to-batch variability. Increasing regulatory pressure to limit use of animal serum has been issued and serum-free, xeno-free, and chemically defined media (S&XFM-CD) is encouraged to replace serum-containing media (SCM) in the stem cell preparation process. We previously developed a S&XFM-CD for the expansion of umbilical cord-derived MSCs (UCMSCs). Different culture conditions affect the function of MSCs, which may further affect the therapeutic efficiency and mechanisms of action. In this study, we compared the therapeutic effect and mechanism of UCMSCs in S&XFM-CD (UCMSC^S&XFM−CD^) in experimental colitis with those in SCM (UCMSC^SCM^). UCMSC^S&XFM−CD^ exhibited better therapeutic effects than UCMSC^SCM^ by body weight, disease activity index, and histological colitis score. UCMSC^S&XFM−CD^ or UCMSC^SCM^ migrated to the inflammation site of injured colon, but exhibited low levels of recruitment and persistence. Systemic depletion of endogenous macrophages impaired the therapeutic effects of UCMSC^SCM^ and UCMSC^S&XFM−CD^. Furthermore, UCMSC^S&XFM−CD^ more markedly promoted intestinal macrophage polarisation from M1 to M2 phenotype to produce higher levels of IL-10 and lower levels of TNF-α in colon tissue than UCMSC^SCM^, while a higher level of IL-4 was produced in UCMSC^SCM^-treated group. UCMSC^S&XFM−CD^ cocultured with RAW264.7 cells in a transwell system promoted the release of TSG-6 and IL-6, whereas UCMSC^SCM^ increased PGE_2_ levels. Taken together, we demonstrated that UCMSCs in S&XFM-CD exhibited improved therapeutic effects with altered cytokine secretion in an experimental acute colitis model.

## Introduction

Inflammatory bowel disease (IBD) is a group of intestinal non-specific inflammatory diseases that mainly includes ulcerative colitis and Crohn's disease. Traditional methods and drugs for IBD treatment frequently cause serious side effects and promote treatment resistance. Therefore, exploring alternative treatment options is urgently required in the clinic (Verstockt et al., 2018). Mesenchymal stromal cells (MSCs) have demonstrated great potential as a feasible and effective strategy in experimental models of IBD (Conklin et al., 2017; Ciccocioppo and Corazza, 2018). However, several preclinical studies have shown that only a low percentage of implanted MSCs can home to the injured tissue and survive *in vivo*, suggesting that the therapeutic action is unlikely to be due to replacement of diseased tissue (Wang et al., 2016; Lopez-Santalla et al., 2017). Indeed, we (Ma et al., 2019b) and others (Barnhoorn et al., 2018; Markovic et al., 2018) have demonstrated that the immunosuppressive characteristics of MSCs provide the theoretical grounds for MSCs therapy in experimental IBD models. Recently, MSCs have also been reported to recruit macrophages to alleviate experimental colitis (Liu et al., 2015). Further research shows that the administration of MSCs ameliorates colitis by decreasing the number of total and M1 macrophages (Park et al., 2018) or increasing the percentage of M2 macrophages in the colon (Song et al., 2017b).

Despite an increasing number of studies showing the benefit of MSCs in preclinical IBD models (Markovic et al., 2018), MSCs involved in these studies are cultured in a medium supplemented with foetal bovine serum (FBS), which has predominantly been used for clinical-grade manufacturing of MSCs (Phinney et al., 2019). FBS is an animal-derived product and is associated with several problematic issues. For example, FBS bears serious safety concerns of transmitting unknown viruses, mycoplasma, prions, or adventitious zoonotic agents. It has been reported that 20–50% of FBS in the market is virus-positive (van der Valk et al., 2018). In addition, FBS could potentially induce undesirable immunologic reactions. Early studies have shown that MSCs grown in FBS-supplemented medium carry a certain amount of FBS proteins (7–30 mg/100 million MSCs) (Jeffrey et al., 2004), which potentially trigger undesirable immunologic reactions (Owens et al., 2016). Furthermore, the exact composition of FBS is unknown and some of these components may be harmful to MSCs growth and cause an unstable transcriptional profile in MSCs (Shahdadfar et al., 2005). Finally, FBS has seasonal and geographical lot-to-lot variability, which could ultimately lead to variability of MSC characteristics and limit the reproducibility of MSC products.

The usage of FBS may ultimately prevent the application of *ex vivo* cultured MSCs in a clinical setting. Increasing regulatory pressure to limit the use of FBS in cell culture products has been issued (van der Valk et al., 2018). According to the guidelines for quality control and preclinical studies of stem cell preparation in China, animal serum should be avoided as much as possible and serum-free, xeno-free, and chemically defined media (S&XFM-CD) is encouraged to replace serum-containing media (SCM) in the stem cell preparation process. Notably, some studies show that several commercially available S&XFM-CD allow for isolation and expansion of MSCs (Corotchi et al., 2013; Simoes et al., 2013; Devito et al., 2014; Swamynathan et al., 2014; Badraiq et al., 2015; Ma et al., 2019a). However, no studies have yet evaluated the effects of MSCs cultured in S&XFM-CD in ulcerative colitis. We have previously developed a S&XFM-CD for the culture of MSCs derived from umbilical cord (UCMSCs) that contains hormones, nutrients, minerals, and growth factors (see Patent No. CN. ZL201210350602.0 and Wu et al., 2016). Moreover, we further confirmed the immunosuppressive effect of UCMSCs in S&XFM-CD on experimental colitis (Ma et al., 2019b). Growing evidence supports that different culture conditions affect the function of cells (Liu et al., 2018; Yoshida et al., 2018; Kong et al., 2019), which may further affect the therapeutic efficiency and mechanisms of action. We reason that S&XFM-CD might impact the therapeutic mechanisms and effects of UCMSCs on IBD. Thus, our study aimed to assess the therapeutic efficacy of UCMSCs in S&XFM-CD in the treatment of IBD and examine its therapeutic mechanisms.

## Materials and Methods

### Ethics Statement

This study was approved by the Ethics Committee of the Beijing Friendship Hospital affiliated with the Capital Medical University (authorization no. 17-2031). All protocols for collecting and processing human umbilical cord samples were approved by the Ethics Committee of Beijing Friendship Hospital affiliated with the Capital Medical University (authorization no. 2017-P2-179-02) with informed maternal consent.

### Preparation of UCMSCs

Umbilical cord samples were collected from healthy full-term pregnant women (age range: 23–31 years, mean: 26 years). UCMSCs were isolated and cultured as described previously (Mu et al., 2018). Briefly, the umbilical vessels were manually removed. Wharton's jelly was minced and digested with an enzyme cocktail at 37°C for 60 min. The digested mixture was passed through a 70 μM mesh and plated in S&XFM-CD or SCM (10% FBS-supplemented medium) at 37°C and 5% CO_2_. The formulation of S&XFM-CD including basal medium and xeno-free defined supplement was showed in Supplementary Table 1, and S&XFM-CD was prepared as described previously (Wu et al., 2016). UCMSCs were selected by adherence to plastic culture plates after 5 days, and passaged at a density of 3,000 cells/cm^2^ when reached 90% confluence. UCMSCs from seven independent donors (*n* = 7) at passage 5 were used for the subsequent experiments in this study.

### Colitis Induction and Treatment

Acute colitis was induced in male C57BL/6 mice aged 6–8 weeks with 2.5% dextran sulphate sodium (DSS, MP Biochemicals, China) in drinking water for 7 consecutive days (Fuenzalida et al., 2016) unless the application of humane endpoint (severe bleeding) was needed. We intraperitoneally injected 1 × 10^6^ UCMSCs in S&XFM-CD or SCM (UCMSC^S&XFM−CD^ and UCMSC^SCM^, respectively) in 100 μL phosphate buffer saline (PBS) into each mouse and monitored their body weight daily. Mice receiving DSS-free water were used as controls (naive). Each experiment was repeated with UCMSCs obtained from different donors, and seven mice were analysed in each experimental group. The disease activity index (DAI) was calculated by combined assessment of weight loss, stool consistency, and bleeding severity (Supplementary Table 2). At the indicated time points, mice were sacrificed and the colon was collected. The entire colon was surgically separated from the cecum to the anus and the colon length was measured.

### UCMSCs Labelling and Imaging

UCMSCs were labelled with the fluorescent dye CM-Dil (Life Technologies, USA) according to the manufacturer's instructions before transplantation. Briefly, UCMSC^S&XFM−CD^ or UCMSC^SCM^ were incubated (37°C, 5 min; 4°C, 15 min) with 2 μg/mL CM-DiI, washed twice with PBS, and injected intraperitoneally into mice on day 0. Mice were sacrificed on day 3 and 10 and 5-μm-thick colon cryosections were made to investigate cell migration *in vivo*. The labelled UCMSCs were observed by fluorescence microscopy.

### *In vivo* Depletion of Macrophages

Mice were fed by drinking water with 2.5% DSS for 7 consecutive days to establish the model as described above, and received 200 μL of dichloromethylene diphosphonate (Cl_2_MDP) liposomes (FormuMax Scientific, Northern California, USA) via intravenous injection once every three days (Hunter et al., 2010) and 24 h prior to and following intraperitoneally injection of UCMSC^S&XFM−CD^, UCMSC^SCM^, or PBS.

### Histological Evaluation

The colon tissues were fixed in 4% paraformaldehyde, serially dehydrated, and embedded in paraffin. The 5-μm-thick sections were collected and stained with haematoxylin and eosin (H&E) for light microscopy. Histological score was calculated by 2 blinded trained pathologists with a combined evaluation of epithelial damage, loss of crypts, and infiltration of inflammatory cells (Supplementary Table 3).

### Flow Cytometry

The colon tissues were digested with 0.1% collagenase type 1 and 0.05% trypsin (Sigma-Aldrich, USA) for 30 min at 37°C. The cell suspensions were passed through a 70 μm cell strainer, collected, and incubated with CD45-PE-cy5, F4/80-FITC, CD86-PE, and CD206-PE (Santa Cruz Biotechnology, USA). Then, the cells were washed and analysed using flow cytometry with a FACS Calibur (BD Biosciences, USA). The gate was set on the CD45^+^ population, and surface markers were further analysed in this gate using Flowjo program (Tree Star, Ashland, OR, USA).

### MSCs/Macrophage Co-cultures

3.5 × 10^5^ RAW264.7 cells (American Type Tissue Collection, Manassas, USA) were seeded in the upper chamber of the transwell insert, followed by lipopolysaccharide (LPS, 100 ng/mL) and interferon-γ (IFN-γ, 10 ng/mL) treatment for 12 h and then cocultured with 3.5 × 10^4^ UCMSC^SCM^ or UCMSC^S&XFM−CD^, which were seeded in the lower chamber for 24 h as described previously (Song et al., 2017b). UCMSCs from seven independent donors were used in this study (*n* = 7), and each sample is repeated three times.

### Quantitative Real-Time Polymerase Chain Reaction

Total RNA was extracted from colon tissue, RAW264.7 cells, and UCMSCs using TRIzol reagent (Invitrogen, USA) and reversely transcribed to cDNA with the QuantiTect Reverse Transcription Kit (Qiagen, Germany). Quantitative real-time polymerase chain reaction (qRT-PCR) was performed using a Platinum SYBR Green PCR Mix (Invitrogen, USA) and a 7700 Sequence Detector (Applied Biosystems, USA). The PCR cycling conditions were 94°C for 3 min, 40 cycles at 94°C for 30 s, 62°C for 30 s, and 72°C for 30 s. The primers are shown in Supplementary Table 4. The mRNA expressions of each gene were analysed and normalised to the expression of glyceraldehyde 3-phosphate dehydrogenase (GAPDH) using the 2^−ΔΔ*Ct*^ method.

### Cytokine and Indoleamine 2,3-Dioxygenase Activity Assay

Secreted protein levels of prostaglandin E_2_ (PGE_2_), interleukin (IL)-6, and tumour necrosis factor-α-induced protein 6 (TSG-6) were determined in co-cultured supernatants using specific cytokine enzyme-linked immunosorbent assay (ELISA) kits (R&D Systems, USA) as per manufacturer's instructions. Indoleamine 2,3-dioxygenase (IDO) activity, determined as kynurenine concentration, was detected using a spectrophotometric assay as described previously (Vasandan et al., 2016). Briefly, 30% trichloroacetic acid was added to the collected supernatants, and centrifuged at 8,000 g for 5 min. 85 μL of the supernatant was transferred to 96-well plates, and 85 μL of 1% Ehrlich reagent was added and incubated for 10 min. The absorbance was measured at 490 nm.

### Statistical Analysis

The data were represented as mean ± standard deviation (SD). A Student's *t*-test was applied to calculate the differences between two groups while a one-way analysis of variance (ANOVA) followed by Tukey's multiple comparisons test was applied for comparison among multiple groups. *P* < 0.05 was considered statistically significant as indicated in each case (^*^indicates *P* < 0.05, ^**^indicates *P* < 0.01 and ^***^indicates *P* < 0.001). Statistical calculations were performed with SPSS 17.0.

## Results

### UCMSC^S&XFM-CD^ Was More Effective Than UCMSC^SCM^ in Alleviating DSS-Induced Colitis

**Figure 1:**
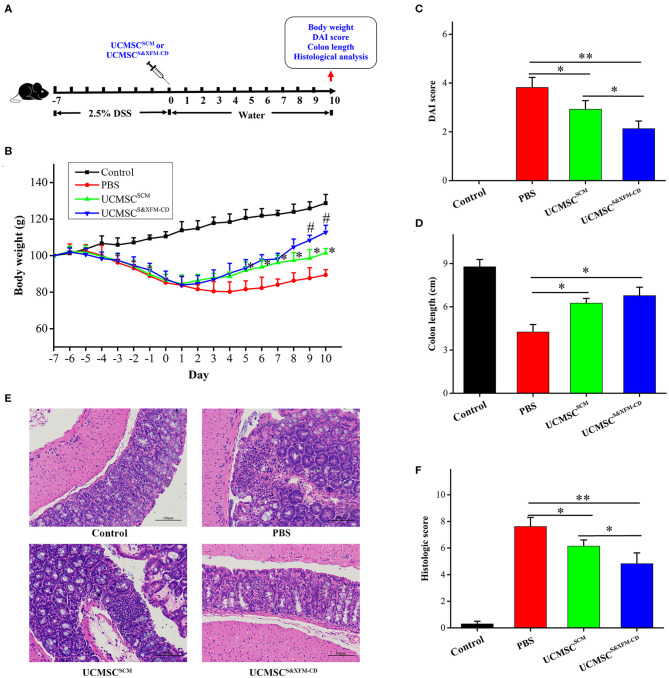
UCMSC^S&XFM−CD^ exhibited better therapeutic effects in a mouse model of acute colitis than UCMSC^SCM^. **(A)** Experimental design of DSS-induced acute colitis. UCMSC^S&XFM−CD^ or UCMSC^SCM^ were injected intraperitoneally and mice were sacrificed on day 10. **(B)** Body weight loss over time (*n* = 7 mice/group, each mouse was transplanted with UCMSCs from a different donor). Significance was analysed using one-way ANOVA followed by Tukey's multiple comparisons test for multiple group comparisons. *p < 0.05 vs. PBS group, ^#^*P* < 0.05 vs. UCMSC^SCM^. **(C)** DAI scores on day 10. **(D)** Disease-related shortening of the colon. **(E)** Representative images of H&E stain of colons on day 10. Scale bars = 100 μm. **(F)** Histological scores on day 10 (*n* = 5 mice/group, each mouse was transplanted with UCMSCs from a different donor). Significance was analysed using one-way ANOVA followed by Tukey's multiple comparisons test for multiple group comparisons. *p < 0.05 and **p < 0.01.

To assess the therapeutic effect of UCMSC^S&XFM−CD^ and UCMSC^SCM^, acute colitis was induced by DSS. UCMSC^S&XFM−CD^ or UCMSC^SCM^ were injected intraperitoneally and mice were sacrificed on day 10 (Figure 1A). UCMSC^S&XFM−CD^ exhibited a rapid recovery of weight loss on days 5–10 compared with PBS and more rapid recovery on days 9 and 10 compared with UCMSC^SCM^ (Figure 1B). The administration of UCMSC^SCM^ or UCMSC^S&XFM−CD^ showed lower DAI scores on day 10 compared with PBS-treated mice and UCMSC^S&XFM−CD^ administration maintained lower DAI scores compared with UCMSC^SCM^ (Figure 1C). We next measured the colon length on day 10 and found that the colon lengths were significantly increased in UCMSC^SCM^- or UCMSC^S&XFM−CD^-treated groups compared with PBS treatment, but no significant differences were observed between UCMSCs-treated groups (Figure 1D). In addition, UCMSC^S&XFM−CD^ was more effective in the amelioration of colon damage than UCMSC^SCM^ as indicated by H&E staining and histopathological scoring (Figures 1E,F). These results suggest that both UCMSC^SCM^ and UCMSC^S&XFM−CD^ alleviated DSS-induced colitis, of which the latter was more effective.

### UCMSC^S&XFM-CD^ and UCMSC^SCM^ Exhibited Low Levels of Recruitment and Persistence in the Injured Site

**Figure 2:**
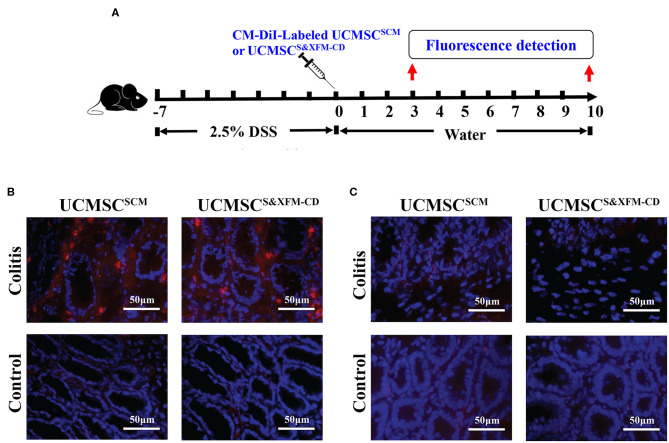
UCMSC^S&XFM−CD^ and UCMSC^SCM^ exhibited low levels of recruitment and persistence in the injured colon of acute colitis mouse model. **(A)** Experimental design for determination of UCMSCs migration to the injured site of the colon. UCMSC^S&XFM−CD^ or UCMSC^SCM^ labelled with CM-DiI were injected intraperitoneally and detected in the colon on days 3 and 10 acute colitis by fluorescence evaluation (*n* = 5 mice/group, each mouse was transplanted with UCMSCs from a different donor). Mice receiving DSS-free water (without colitis) were used as controls. **(B)** Representative images of colonic fluorescence on day 3. UCMSC^S&XFM−CD^ or UCMSC^SCM^ (red) migrated to the inflamed colon of colitis animals on day 3, but not in the non-inflamed colon of control mice. Nucleuses were stained with 4′,6-diamidino-2-phenylindole (DAPI, blue). **(C)** Representative images of colonic fluorescence on day 10. No labelled UCMSC^S&XFM−CD^ or UCMSC^SCM^ were observed in both colitis and control animals. Scale bars = 50 μm.

To determine whether UCMSC^S&XFM−CD^ or UCMSC^SCM^ could migrate to the injured site of the colon with acute colitis, 1 × 10^6^ UCMSC^S&XFM−CD^ or UCMSC^SCM^ labelled with CM-DiI were injected intraperitoneally and detected in the colon on days 3 and 10 (Figure 2A). As shown in Figure 2B, CM-Dil could be detected in the inflamed colon of acute colitis mice at 3 days post-cell transplantation, but no fluorescence was found in the colon of normal mice. Moreover, no fluorescence was detected in the colon of acute colitis or normal mice at day 10 (Figure 2C). These results suggest that intraperitoneally injected UCMSC^S&XFM−CD^ or UCMSC^SCM^ could migrate to the inflammation site of the injured colon, but both UCMSCs exhibited low levels of recruitment and persistence.

### Depletion of Macrophages Impaired the Therapeutic Effects of UCMSC^SCM^ and UCMSC^S&XFM-CD^

**Figure 3:**
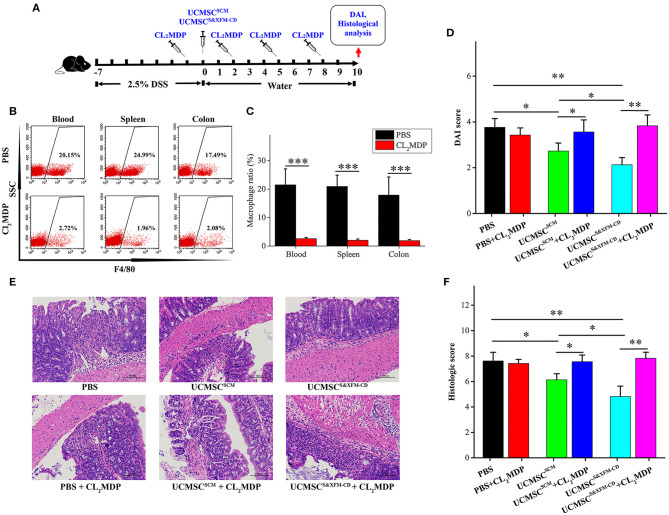
Systemic depletion of endogenous macrophages impaired the therapeutic effects of UCMSC^SCM^ and UCMSC^S&XFM−CD^ treatment. **(A)** Macrophage depletion protocol using Cl_2_MDP liposomes in DSS-induced acute colitis. UCMSC^S&XFM−CD^ or UCMSC^SCM^ were injected intraperitoneally and mice were sacrificed on day 10. **(B)** Representative flow cytometry plots and **(C)** proportions of F4/80^+^ macrophages in the spleen, blood, and colon from Cl_2_MDP- and PBS-treated animals on day 3 (*n* = 5 mice/group, each mouse was transplanted with UCMSCs from a different donor). Significance was analysed using Student's *t*-test for two group's comparison. ***P < 0.001. **(D)** DAI scores on day 10 (*n* = 5 mice/group, each mouse was transplanted with UCMSCs from a different donor). Significance was analysed using Student's *t*-test for two group's comparison. *p < 0.05 and **p < 0.01. **(E)** Representative images of H&E stain of colons. Scale bars = 100 μm. **(F)** Histological scores on day 10 (*n* = 5 mice/group, each mouse was transplanted with UCMSCs from a different donor). Significance was analysed using Student's *t*-test for two group's comparison. *p < 0.05 and **p < 0.01.

To investigate whether the macrophages were involved in the role of UCMSCs in different media, we intravenously injected Cl_2_MDP liposomes once every 3 days and 24 h prior to and following intraperitoneally injection of UCMSC^S&XFM−CD^ or UCMSC^SCM^ (Figure 3A). As expected, intravenous injection of Cl_2_MDP liposomes markedly reduced macrophage proportions in blood, spleen, and colon (Figures 3B,C). Cl_2_MDP itself did not aggravate colitis as we observed no significant difference in DAI and histopathological scoring between the PBS-treated group and the PBS and Cl_2_MDP-treated group (Figures 3D–F). However, additional Cl_2_MDP liposomes attenuated the benefits of UCMSC^S&XFM−CD^ or UCMSC^SCM^ (Figures 3D–F). Furthermore, UCMSC^S&XFM−CD^ or UCMSC^SCM^ showed no further therapeutic effect when administered in conjunction with Cl_2_MDP (Figures 3D–F). Collectively, our results suggest that depletion of macrophages impaired the benefits of UCMSC^S&XFM−CD^ or UCMSC^SCM^, indicating that the therapeutic effects of MSCs depend on macrophages.

### UCMSC^S&XFM-CD^ Promoted More Intestinal Macrophage Polarization From M1 to M2 Phenotype Than UCMSC^SCM^ *in vivo*

**Figure 4:**
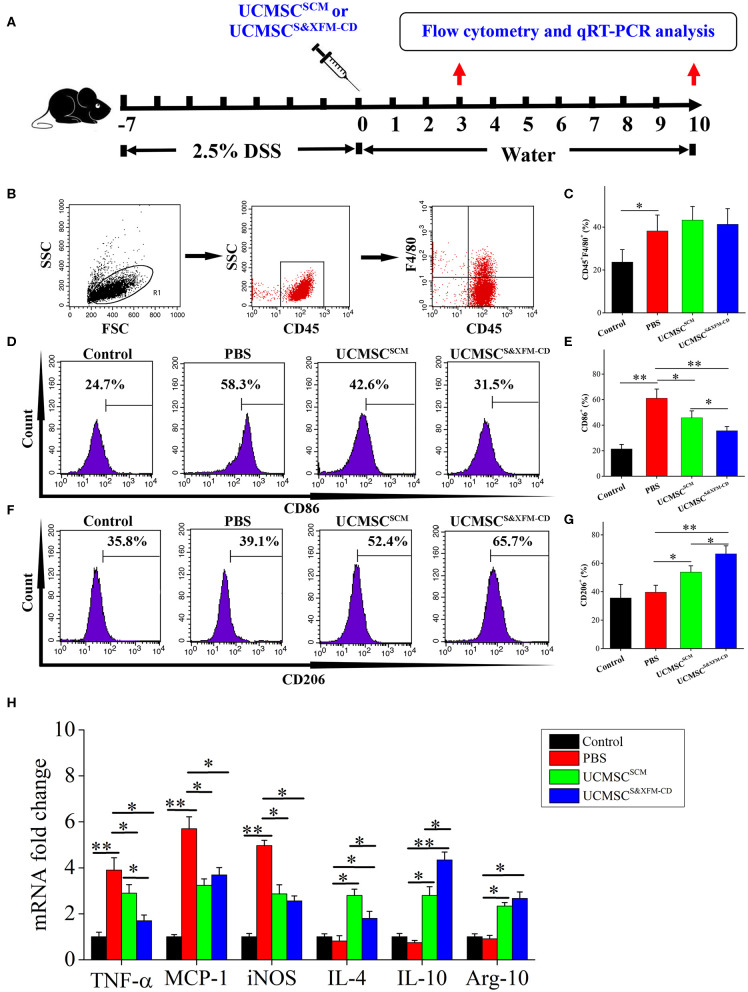
UCMSC^S&XFM−CD^ more markedly promoted intestinal macrophage polarization from M1 to M2 phenotype than UCMSC^SCM^. **(A)** Experimental design for the infiltration and polarization of macrophages in the colon. Acute colitis was induced and UCMSC^S&XFM−CD^ or UCMSC^SCM^ were injected intraperitoneally and the ratio of CD45^+^, F4/80^+^, CD86^+^, and CD206^+^ cells was analysed by flow cytometry and qRT-PCR on days 3 and 10. **(B)** Colonic macrophage gating strategy. Proportions of **(C)** F4/80^+^, **(D)** CD86^+^ M1, **(E)** CD86^+^, **(F)** CD206^+^ M2, and **(G)** CD206^+^ macrophages in the colon on day 3 (*n* = 7 mice/group, each mouse was transplanted with UCMSCs from a different donor). **(H)** qRT-PCR analysis of gene expression in colon on day 3 (*n* = 7 mice/group, each mouse was transplanted with UCMSCs from a different donor). Significance was analysed using one-way ANOVA followed by Tukey's multiple comparisons test for multiple group comparisons. *p < 0.05 and **p < 0.01.

To further investigate whether administering UCMSC^SCM^ or UCMSC^S&XFM−CD^ affect the infiltration and polarization of macrophages in the injured area of the colon with acute colitis, 1 × 10^6^ UCMSC^S&XFM−CD^ or UCMSC^SCM^ were injected intraperitoneally and the ratio of CD45^+^, F4/80^+^, CD86^+^, and CD206^+^ cells was analysed by flow cytometry and qRT-PCR on days 3 and 10 (Figure 4A). Flow cytometric analysis revealed that the proportion of F4/80^+^ macrophages was markedly increased on day 3 in PBS-treated colitis mice compared with normal mice, but was not changed after UCMSC^SCM^ or UCMSC^S&XFM−CD^ treatment (Figures 4B,C). However, further analysis showed that the proportion of CD86^+^ cells representing M1 macrophages was dramatically decreased on day 3 in UCMSC^SCM^-treated mice, which was further exacerbated by UCMSC^S&XFM−CD^ treatment (Figures 4D,E). Conversely, the proportion of CD206^+^ cells representing M2 macrophages was significantly increased in UCMSC^SCM^-treated group and UCMSC^S&XFM−CD^ treatment further promoted this effect (Figures 4F,G). Furthermore, qRT-PCR analysis showed that the expression of M1-related genes, such as tumour necrosis factor alpha (TNF-α), monocyte chemoattractant protein-1 (MCP-1), and inducible nitric oxide synthase (iNOS) in colon tissue were significantly reduced on day 3, whereas M2-related genes, such as IL-4, IL-10, and arginase-1 (Arg-1) were increased in UCMSC^SCM^- or UCMSC^S&XFM−CD^-treated groups. Similarly, UCMSC^S&XFM−CD^ treated group showed higher IL-10 but lower IL-4 and TNF-α levels than UCMSC^SCM^ (Figure 4H). We observed similar trends on day 10 (data not shown). Collectively, these data demonstrate that UCMSC^S&XFM−CD^ markedly promoted intestinal macrophage polarisation from M1 to M2 phenotype to produce different inflammatory factors in colon tissue compared with UCMSC^SCM^.

### UCMSC^SCM^ and UCMSC^S&XFM-CD^ Polarised Macrophages From M1 to M2 Phenotype Through Different Cytokine Secretion *in vitro*

**Figure 5:**
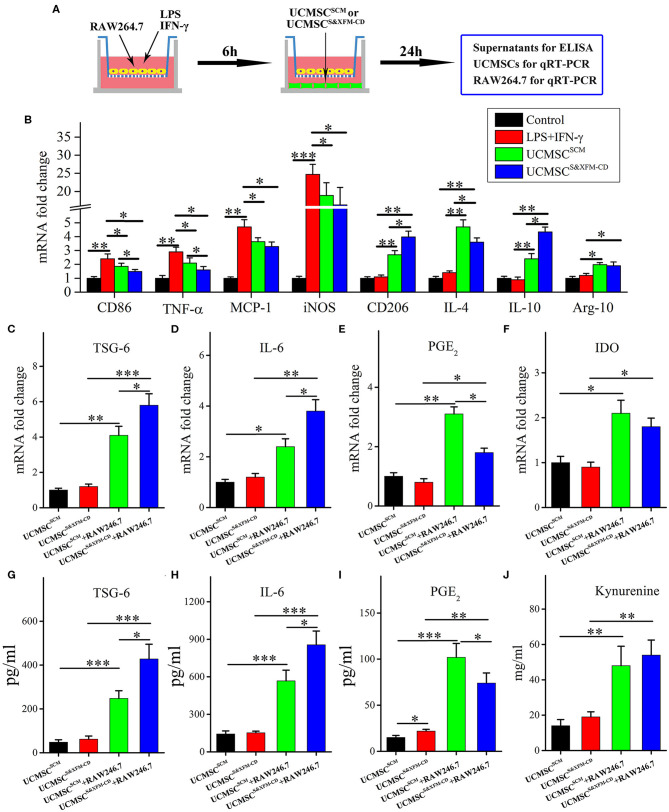
UCMSC^SCM^ and UCMSC^S&XFM−CD^ polarized macrophages from M1 to M2 phenotype *in vitro* through different mechanisms. **(A)** Schematic depiction of the cell coculture system. RAW264.7 cells were seeded in the upper chamber of transwell insert and cocultured with UCMSC^SCM^ or UCMSC^S&XFM−CD^ in the lower chamber. **(B)** mRNA levels of M1- and M2-related markers, pro-inflammatory factors, and anti-inflammatory factors in RAW264.7 cells by qRT-PCR (*n* = 7 independent experiments). **(C–F)** mRNA and **(G–I)** protein levels of TSG-6, IL-6, PGE_2_, and IDO in UCMSCs. **(J)** IDO activity, determined as kynurenine concentration in UCMSCs (*n* = 7 independent experiments). Significance was analysed using one-way ANOVA followed by Tukey's multiple comparisons test for multiple group comparisons. *p < 0.05, **p < 0.01, and ***p < 0.001.

To further better understand the differences in the molecular mechanisms between UCMSC^SCM^ and UCMSC^S&XFM−CD^ on macrophage polarisation, we established a transwell-based coculture system (Figure 5A). UCMSC^S&XFM−CD^ and UCMSC^SCM^ significantly inhibited mRNA upregulation of the M1 marker CD86 and pro-inflammatory factors including TNF-α, MCP-1, and iNOS in the RAW264.7 cells (Figure 5B). Notably, the inhibitory effect of UCMSC^S&XFM−CD^ on TNF-α expression was much greater than UCMSC^SCM^. Conversely, UCMSC^S&XFM−CD^ and UCMSC^SCM^ caused upregulation of the mRNA levels of the M2 markers CD206 and Arg-1 and anti-inflammatory factors, such as IL-4 and IL-10 (Figure 5B). Similarly, UCMSC^S&XFM−CD^ was more capable of upregulating IL-10, but showed decreased IL-4 expression compared with UCMSC^SCM^ (Figure 5B). We also investigated the expression profile of the UCMSCs and found that UCMSC^SCM^ promoted M2 macrophage polarisation by increasing the mRNA expression of TSG-6, PGE_2_, IL-6, and IDO. Moreover, the mRNA expression levels of TSG-6 and IL-6 in UCMSC^S&XFM−CD^ were significantly higher, although the level of PGE_2_ was significantly lower than that in UCMSC^SCM^ (Figures 5C–E). In addition, IDO expression did not differ between the two UCMSCs (Figure 5F). Similar trends were observed at the protein level of TSG-6, IL-6, PGE_2_, and IDO by ELISA analysis (Figures 5G–J). Taken together, these data indicate that UCMSC^SCM^ and UCMSC^S&XFM−CD^ significantly polarised macrophages from M1 to M2 phenotype through secretion of different cytokine profiles *in vitro*.

## Discussion

In this study, our data showed that UCMSC^S&XFM−CD^ exhibited better therapeutic efficacy in an acute colitis mouse model compared with UCMSC^SCM^. It has been reported that UCMSCs can be manipulated *in vitro* by preconditioning in SCM and deconditioning in serum-free medium, leading to enhanced immunosuppressive and therapeutic effects on IBD (Yang et al., 2018). Although the components of serum-free media in the previous studies are different from ours, the reported results are consistent with our findings, which indicates that S&XFM-CD enhances the effectiveness of UCMSCs for the treatment of IBD and may represent an attractive alternative to FBS for culturing clinical-grade UCMSCs.

Some studies show that intraperitoneally injected MSCs disseminate to heart, lung, liver, spleen, and kidney, but do not migrate to the colon (Song et al., 2017b). However, other studies have confirmed that intraperitoneal but not intravenous MSCs could migrate to the inflammatory site of the injured colon (Castelo-Branco et al., 2012; Lee et al., 2018; Lopez-Santalla et al., 2018). Our data showed that CM-Dil-labeled UCMSC^SCM^ and UCMSC^S&XFM−CD^ were only transiently recruited to the injured colon and both UCMSCs exhibited low levels of recruitment and persistence in experimental colitis. Due to the sensitivity of fluorescence imaging, a low number of MSCs may remain undetected. Therefore, we tracked colon colonization by MSCs using qRT-PCR as described previously (Song et al., 2017b). The presence of UCMSCs existence was detected at day 3, but gradually decreased over time and both UCMSC^SCM^ and UCMSC^S&XFM−CD^ were no longer present in the inflamed colon after day 3 (data not shown). The loss of colonic MSCs may be attributed to many causes, such as washout, cell death, or even rejection by the innate immune system. Although MSCs were initially proposed for as a therapeutic tool based on their differentiation capability, the lack of cell engraftment or differentiation at the site of injury has led to suggestions that MSCs may exert their therapeutic effects mainly through paracrine signalling (Shi et al., 2018). In this study, the intraperitoneal injection of UCMSC^S&XFM−CD^ or UCMSC^SCM^ significantly ameliorated the severity of experimental colitis. Our previous study also shows the therapeutic effect of exosomes from UCMSCs in S&XFM-CD on experimental colitis (Ma et al., 2019b), thus we postulate that the therapeutic benefits of intraperitoneally injected UCMSC^SCM^ or UCMSC^S&XFM−CD^ stem from immunomodulatory mechanisms and are mediated by indirect paracrine factors rather than direct cell-to-cell interactions. This hypothesis is consistent with the current view that MSCs exerted its benefit via its paracrine effect (Khubutiya et al., 2014).

Macrophages play a critical role in the modulation of colon inflammation after IBD (Isidro and Appleyard, 2016). Therefore, we investigated the role of macrophages in UCMSC^SCM^ or UCMSC^S&XFM−CD^ therapy. Our data showed that the depletion of macrophages abolished the beneficial effects of both UCMSC^SCM^ and UCMSC^S&XFM−CD^ transplantation in acute colitis, consistent with results in animal models of other diseases including allergic asthma (Mathias et al., 2013), myocardial infarction (Ben-Mordechai et al., 2013; Wang et al., 2015), and liver injury (Ghanem et al., 2019). Thus, we confirmed the hypothesis that the protective effects of UCMSCs on acute colitis are mediated by macrophages independent of culture conditions.

MSCs polarize macrophages from pro-inflammatory M1 to anti-inflammatory M2 to exert an immunosuppressive and therapeutic effect (Zheng et al., 2015; Mao et al., 2017). Therefore, we examined the potential effects of UCMSC^SCM^ or UCMSC^S&XFM−CD^ on macrophage quantity and subpopulations *in vivo*. Our results suggested that UCMSC^SCM^ or UCMSC^S&XFM−CD^ increased the proportion of total macrophages in the colon, which was consistent with some studies (Liu et al., 2015), although others show macrophage suppression or no significant changes in macrophage populations (Simovic Markovic et al., 2016; Song et al., 2017b; Park et al., 2018). We speculate that the inconsistencies may be due to different detection times or methods. Interestingly, recent studies have shown that macrophage proportions are not altered when exosomes from MSCs are administrated at the same time using the same methods (Liu et al., 2019). This indicates that there are functional differences between exosomes and MSCs. Our analysis showed that UCMSC^SCM^ or UCMSC^S&XFM−CD^ polarised macrophages from proinflammatory M1 to anti-inflammatory M2 macrophages, which dampen intestinal inflammation. Similar findings have been reported previously (Song et al., 2017a; Liu et al., 2019), although other studies have shown contradicting conclusions (Song et al., 2017b; Park et al., 2018). One study shows that the administration of MSCs ameliorates colitis by decreasing the proportion of M1 macrophages population, but found no significant change in M2 macrophages (Park et al., 2018) and conversely another study demonstrates that this effect is caused by an increase in the percentage of M2 macrophages without affecting M1 macrophages (Song et al., 2017b). These discrepancies may be related to different times of cell transplantation. We observed that MSCs cultured in S&XFM-CD further enhanced macrophage polarization from proinflammatory M1 to anti-inflammatory M2 macrophages and was consistent with previous reports by Yoshida and colleagues who used a different serum-free medium (Yoshida et al., 2018). It has been reported that the immunological paradigm of M1/M2 dichotomy following macrophage polarisation is unclear in humans due to key differences in macrophage biology between human and mouse (Na et al., 2019). There is a continuum between M1-like and M2-like macrophages where boundaries are still unclear. A current challenge in the study of macrophage phenotypes is that some markers used to identify M1 and M2 macrophages in mice cannot be directly applied to human subsets (Watanabe et al., 2019). For example, iNOS and Arg1 are well-established markers for mouse M1 and M2 macrophages, respectively, but their significance in the human subsets has not been defined. This limitation likely explains emerging literature in which the data collected in mice and humans reveal macrophage phenotypes that are inconsistent with the M1/M2 paradigm (Hine and Loke, 2019).

**Figure 6:**
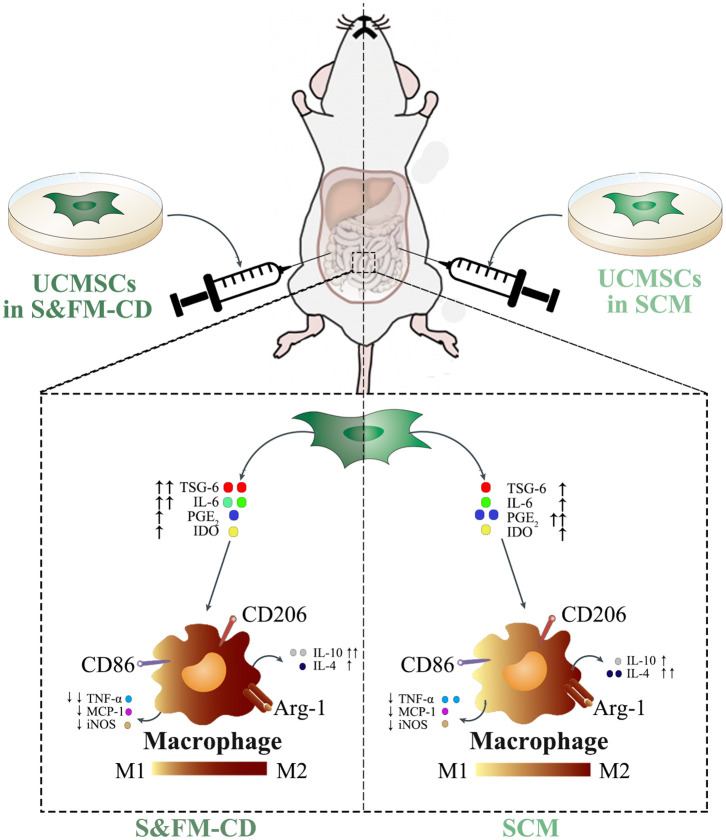
UCMSC^SCM^ and UCMSC^S&XFM−CD^ dampened DSS-induced acute colitis though polarisation of macrophages from M1 to M2 phenotype, mediated via different mechanisms. In response to the proinflammatory environment, UCMSC^SCM^ or UCMSC^S&XFM−CD^ secreted immunomodulatory mediators including PGE_2_, TSG-6, IL-6, and IDO. These mediators inhibited the polarisation of M1 macrophages and the production of pro-inflammatory cytokines (TNF-α, MCP-1, and iNOS). In addition, they promoted the polarisation of M2 macrophages and the production of anti-inflammatory cytokines (IL-4 and IL-10), which lead to the attenuation of DSS-induced colon inflammation and injury. However, culture condition altered the mechanisms employed by the UCMSCs. While UCMSC^SCM^ produced PGE_2_ and increased the release of IL-4 to promote intestinal macrophage polarization to an M2 phenotype, UCMSC^S&XFM−CD^ secreted TSG-6 and IL-6 to achieve this more effectively and further increased IL-10 and reduced TNF-α compared with UCMSC^SCM^. This culminated in the improved therapeutic effects of UCMSC^S&XFM−CD^ in DSS-induced colitis.

Understanding the molecular mechanisms involved in the crosstalk between MSCs and macrophages will contribute to the optimal use of MSCs in clinical practice. Our data demonstrated that UCMSC^S&XFM−CD^ more markedly promoted intestinal macrophage polarization from M1 to M2 phenotype to produce higher levels of IL-10 and lower levels of TNF-α in colon tissue compared with UCMSC^SCM^, similar to previous reports (Simovic Markovic et al., 2016; Song et al., 2017b; Liu et al., 2019). To our surprise, UCMSC^S&XFM−CD^ treatment also lowered the expression of IL-4. This suggests that UCMSCs may be involved in multiple inflammatory processes and that UCMSCs in different media employ other pathways. MSC-mediated macrophage polarization has been demonstrated in various inflammatory diseases and is regulated by several mediators secreted by MSCs, such as PGE_2_ (Ylostalo et al., 2012; Vasandan et al., 2016; Park et al., 2018), IL-6 (Deng et al., 2015; Xie et al., 2015), TSG-6 (Shin et al., 2016; Di et al., 2017; Song et al., 2017b; Yoshida et al., 2018), and IDO (Francois et al., 2012; Lee et al., 2016). We confirmed that UCMSC^S&XFM−CD^ cocultured with RAW264.7 cells in a transwell system promoted M2 macrophage polarization by decreasing the release of PGE_2_ and increasing TSG-6 and IL-6. PGE_2_ and TSG-6 patterns were in line with data described by Yoshida et al. (2018), but the increase in IL-6 was inconsistent, which may be related to the different components of the serum-free medium. Taken together, these data further indicate that UCMSCs in different media mediate their therapeutic effects through different cellular mechanisms in an acute colitis mouse model. In our case, UCMSC^S&XFM−CD^ moderated inflammation mainly through TSG-6 and IL-6-dependent mechanisms while UCMSC^SCM^ primarily utilised the PGE_2_ pathway (Figure 6).

In conclusion, this study found that intraperitoneal administration of UCMSC^S&XFM−CD^ exhibited better therapeutic effects than UCMSC^SCM^ for the treatment of IBD. Moreover, UCMSC^S&XFM−CD^ more markedly dampened intestinal inflammation by enhancing macrophage polarisation from proinflammatory M1 to anti-inflammatory M2. Mechanistically, we observed that UCMSC^S&XFM−CD^ and UCMSC^SCM^ mediated their therapeutic effects through different pathways. In addition to these notable effects, S&XFM-CD was useful for culturing UCMSCs due to several advantages over SCM including safety, efficacy, consistency, and reproducibility. In conclusion, our results suggest that the usage of S&XFM-CD will accelerate the clinical translation of UCMSCs and strengthen the therapeutic potential of UCMSCs in the treatment of IBD.

## Data Availability Statement

The datasets analyzed in this article are not publicly available. Requests to access the datasets should be directed to XW, stemcells@foxmail.com.

## Ethics Statement

The studies involving human participants were reviewed and approved by Ethics Committee of Beijing Friendship Hospital affiliated to Capital Medical University. The patients/participants provided their written informed consent to participate in this study. The animal study was reviewed and approved by Ethics Committee of Beijing Friendship Hospital affiliated to Capital Medical University.

## Author Contributions

XW: conception and design, collection and/or assembly of data, data analysis and interpretation, manuscript writing, and final approval of manuscript. DW: collection and/or assembly of data, data analysis and interpretation, manuscript writing, and final approval of manuscript. YM: provision of study material, collection and/or assembly of data, data analysis and interpretation. YZ: provision of study material, collection and/or assembly of data. ZM: conception and design, financial support, manuscript writing, and final approval of manuscript.

## Conflict of Interest

The authors declare that the research was conducted in the absence of any commercial or financial relationships that could be construed as a potential conflict of interest.
