# Supplementary material for: Serum-Free Medium Enhances the Therapeutic Effects of Umbilical Cord Mesenchymal Stromal Cells on a Murine Model for Acute Colitis
Source: Front Bioeng Biotechnol. 2020 Jun 26;8:586. doi: 10.3389/fbioe.2020.00586 (PMC7332562; doi:10.3389/fbioe.2020.00586)
Supplement: Supplementary file 1 [file Table_1.DOCX]

Supplementary Table 1**.** The composition of chemically deﬁned serum-free medium for UCMSCs.

| Components | Company | Concentration |
| --- | --- | --- |
| Iscove's Modified Dubecco's Medium | Life technologies | 17.7 g/L |
| L-glutamine | Life technologies | 5 mM |
| Sodium bicarbonate | Life technologies | 3.024 g/L |
| Recombinant human insulin | Sigma | 10 mg/L |
| Recombinant human transferrin | Sigma | 10 mg/L |
| Recombinant human serum albumin | Sigma | 4 g/L |
| β-mercaptoethanol | Life technologies | 55 µM |
| Chemically defined lipid concentrate | Life technologies | 0.1% |
| MEM Essential amino acids Solution | Life technologies | 2% |
| MEM Non-essential Amino Acid Solution | Life technologies | 1% |
| Vitamins Solution | Life technologies | 1% |
| Trace elements Solution | Corning cellgro | 0.1% |
| Hydrocortisone | Sigma | 50µg/L |
| L-ascorbic acid-2-phosphate | Sigma | 50mg/L |
| Recombinant human ﬁbronectin | Sigma | 5mg/L |
| Progesterone | Sigma | 5µg/L |
| Putrescine | Sigma | 10mg/L |
| Serotonin | Sigma | 2mg/L |
| Recombinant human epidermal growth factor | R&D | 10 ng/ml |
| Recombinant human basic ﬁbroblast growth factor | R&D | 10 ng/ml |
| Recombinant human platelet-derived growth factor | R&D | 10 ng/ml |
| Recombinant human insulin-like growth factor | R&D | 10 ng/ml |

Supplementary Table 2. The disease activity index scoring, based on the clinical sign of colitis

|  | Score Bleeding | Stool consistency | Body weight changes |
| --- | --- | --- | --- |
| 0 | Negative | Formed | With no change/increase |
| 1 | Positive occult blood test | Soft | 1%–5% decrease |
| 2 | Visible bleeding | Loose | 6%–10% decrease |
| 3 | Severe bleeding | Watery | 11%–20% decrease |
| 4 | - | - | More than 20% decrease |

Supplementary Table 3. Colonic Histological Scoring System

| Histological feature | Score | Description |
| --- | --- | --- |
| Epithelial damage | 0 | None |
|  | 1 | 0%–5% loss of epithelium |
|  | 2 | 5%–10% loss of epithelium |
|  | 3 | Over 10% loss of epithelium |
| Loss of crypts | 0 | None |
|  | 1 | 0%–10% loss of crypts |
|  | 2 | 10%–20% loss of crypts |
|  | 3 | Over 20% loss of crypts |
| Infiltration of inflammatory cells | 0 | None |
|  | 1 | Mild (10%) |
|  | 2 | Moderate (25%) |
|  | 3 | Severe (40%) |

Supplementary Table 4**.** Primers used for quantitative real-time PCR analysis.

| **Primer name** | **Primer sequence** | **Efficiency (%)** |
| --- | --- | --- |
| mGAPDH | F: AGTATGTCGTGGAGTCTACTGGTGT  R: AGTGAGTTGTCATATTTCTCGTGGT | 91.3 |
| mTNF-α | F: CCAGGAGAAAGTCAGCCTCCT  R: TCATACCAGGGCTTGAGCTCA | 93.8 |
| miNOS | F: AAAGGAAATAGAAACAACAGGAACC  R: GCATAAAGTATGTGTCTGCAGATGT | 93.4 |
| mMCP-1 | F: CTCACCTGCTGCTACTCATTC  R: TTACGGCTCAACTTCACATTCA | 91.5 |
| mIL-4 | F: CAGCAACGAAGAACACCACAG  R: CGAAAAGCCCGAAAGAGTC | 94.2 |
| mIL-10 | F: TGGCCCAGAAATCAAGGAGC  R: CAGCAGACTCAATACACACT | 92.6 |
| mCD86 | F: GGCCGCACGAGCTTTG  R: CGAGCCCATGTCCTTGATCT | 90.1 |
| mCD206 | F: AACGGAATGATTGTGTAGTTCTAGC  R: TACAGGATCAATAATTTTTGGCATT | 92.3 |
| mArg1 | F: CAGAAGAATGGAAGAGTCAG  R: CAGATATGCAGGGAGTCACC | 92.7 |
| hTSG-6 | F: AAAAACTGGCATTATTGATTATGGA  R: CAGTAGCAGATTTGGTTATCTTCGT | 94.9 |
| hIL-6 | F: AAGCCAGAGCTGTGCAGATGAGTA  R: TGTCCTGCAGCCACTGGTTC | 94.7 |
| hPGE_2_ | F: TGACCAGAGCAGGCAGATGAA  R: CCACAGCATCGATGTCACCATAG | 93.1 |
| hIDO | F: CCTGAGGAGCTACCATCTGC  R: TCAGTGCCTCCAGTTCCTTT | 91.8 |
| hGAPDH | F: CGACCACTTTGTCAAGCTCA  R: AGGGGTCTACATGGCAACTG | 90.3 |
